# A carbon nanotube integrated microfluidic device for blood plasma extraction

**DOI:** 10.1038/s41598-018-31810-x

**Published:** 2018-09-11

**Authors:** Yin-Ting Yeh, Zhong Lin, Si-Yang Zheng, Mauricio Terrones

**Affiliations:** 1 Department of Physics and Center for 2-Dimensional and Layered Materials, The Pennsylvania State University, University Park, PA 16802 USA; 2 Department of Biomedical Engineering, The Pennsylvania State University, University Park, PA 16802 USA; 3 Department of Chemistry, Department of Materials Science and Engineering and Center for Atomically Thin Multifunctional Materials (ATOMIC), The Pennsylvania State University, University Park, PA 16802 USA; 4 Institute of Carbon Science and Technology, Shinshu University, 4-17-1 Wakasato, Nagano, 380-8553 Japan; 5 Department of Materials Science and Engineering & Chemical Engineering, Carlos III University of Madrid, Avenida Universidad 30, 28911 Leganés, Madrid, Spain; 6 IMDEA Materials Institute, Eric Kandel 2, Getafe, Madrid, 28005 Spain

## Abstract

Blood is a complex fluid consisting of cells and plasma. Plasma contains key biomarkers essential for disease diagnosis and therapeutic monitoring. Thus, by separating plasma from the blood, it is possible to analyze these biomarkers. Conventional methods for plasma extraction involve bulky equipment, and miniaturization constitutes a key step to develop portable devices for plasma extraction. Here, we integrated nanomaterial synthesis with microfabrication, and built a microfluidic device. In particular, we designed a double-spiral channel able to perform cross-flow filtration. This channel was constructed by growing aligned carbon nanotubes (CNTs) with average inter-tubular distances of ~80 nm, which resulted in porosity values of ~93%. During blood extraction, these aligned CNTs allow smaller molecules (e.g., proteins) to pass through the channel wall, while larger molecules (e.g., cells) get blocked. Our results show that our device effectively separates plasma from blood, by trapping blood cells. We successfully recovered albumin -the most abundant protein inside plasma- with an efficiency of ~80%. This work constitutes the first report on integrating biocompatible nitrogen-doped CNT (CN_x_CNT) arrays to extract plasma from human blood, thus widening the bio-applications of CNTs.

## Introduction

Carbon nanotubes (CNTs) consist of nanometer-scaled tubules of sp^2^ hybridized carbon atoms. They possess unique thermal, optical, electrical, and mechanical properties. CNTs have also been utilized in various applications^1–4^, including sensors^5–7^, field-effect transistors^8–10^, batteries^11–14^, capacitors/actuators^15,16^, hydrogen storage components^17–19^, field emission devices^20–24^, and composite fillers^25,26^.

Due to recent advances in the CNT synthesis and functionalization, their applications have expanded rapidly in the biological fields^27,28^. For example, the biocompatibility of CNTs can be improved by substitutional doping with nitrogen atoms^29–31^. CNT-based biosensors have also been demonstrated to have an enhanced electrochemical reactivity as a result of their high surface area (10^2^~10^3^ m^2^g^−1^)^32,33^. By integrating the growth of aligned CNTs with microfabrication, a 3-dimensional (3D) filter can be built to capture various types of biomolecules and biomarkers. e.g., tumor cells, bacteria, viruses, nuclei acids, and proteins^34–36^. Through selective growing of aligned CNTs along different micro-patterns, novel filtration devices can be designed for the separation of heterogeneous mixtures, including blood.

Blood is a complex fluid consisting of cells and plasma. This plasma is a bodily fluid containing different types of molecules and ions, e.g., clotting factors, proteins, electrolytes, hormones, enzymes, antibodies, vitamins, sugars, lipids, and minerals. In clinical diagnostics, plasma is vital because it can provide relevant information regarding a patient’s health. It is also noteworthy that blood cells cause background noises during the detection. To achieve effective detection, plasma separation is a critical step. In this context, centrifugation is the conventional route to separate plasma from blood. Although the efficiency is extremely high (>90%), bulky equipment is involved. As an alternative, the development of microfluidic devices provides a miniaturized technology able to separate plasma from blood^37^.

In this paper, we integrated CNT synthesis with microfabrication techniques to construct a CNT-based microfluidic device. This microdevice effectively separates plasma from blood by performing cross-flow filtration. Our work now expands applications of CNTs in point-of-care blood analysis.

## Design and Manufacturing of the Plasma Extraction Device

**Figure 1:**
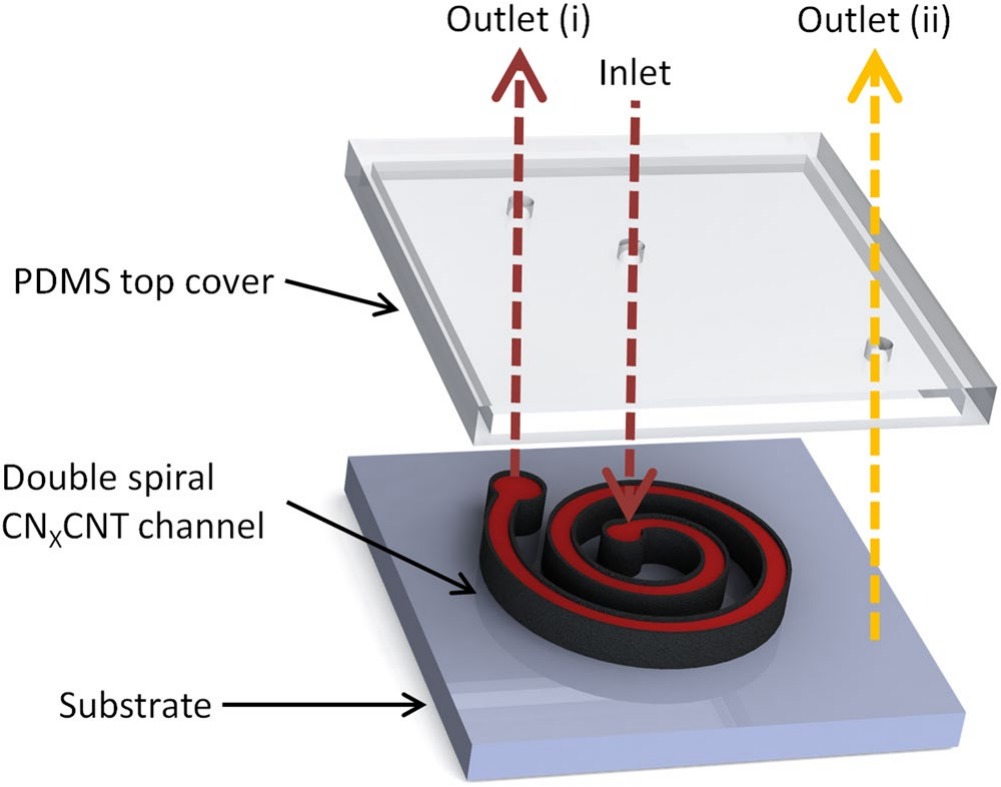
Illustration of microdevice assembly and labels showing sample access ports. Blood is loaded at the inlet and flowed through to the outlet (i). Extracted plasma is collected at the outlet (ii).

We designed a double spiral microdevice to continuously separate plasma from blood by using cross flow filtration as illustrated in Fig. 1. This microdevice was constructed by integrating porous aligned nitrogen doped multi-walled CNT (CN_x_CNT) channel and a polydimethylsiloxane (PDMS) top cover. Inside this microdevice, we constructed a porous microfluidic channel by aligning CN_x_CNTs to form a membrane. The microdevice has one inlet and two outlets (i and ii). Blood samples were then loaded from the inlet port. Human whole blood was obtained from consented donors at the General Clinical Research Center of Penn State, following to an institutional review board–approved protocol. The samples were drawn into 10-mL Ethylenediaminetetraacetic acid (EDTA) K_2_-tubes (Vacutainer; Becton Dickinson). The blood flowing through the device was then collected by the outlet (i). Note that outlet (ii) was connected to a vacuum source to extract and collect the plasma. When blood was transported within the double spiral channel, plasma diffuses through CN_x_CNTs and it is collected at the outlet (ii).

**Figure 2:**
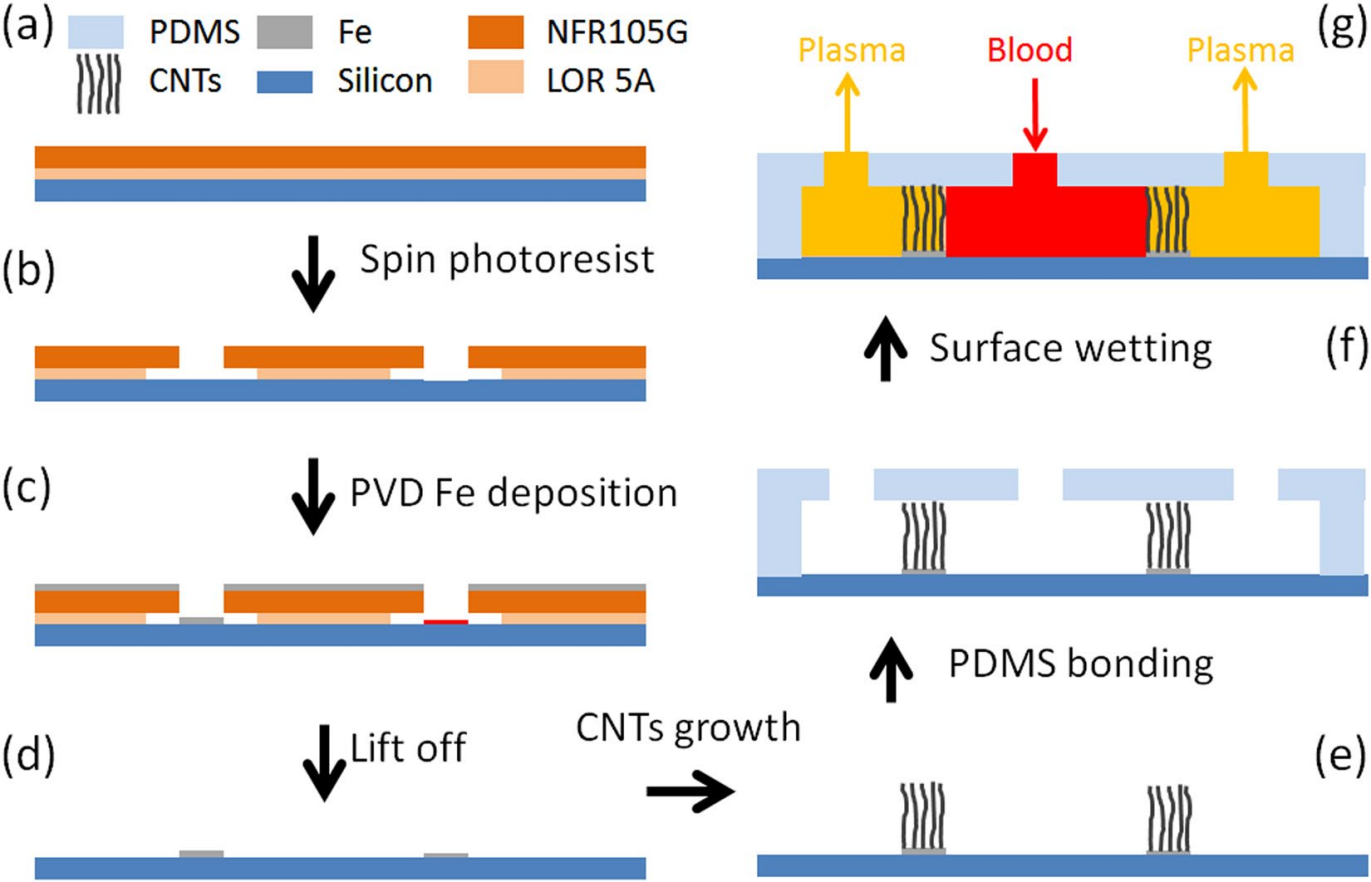
Schematics showing microdevice fabrication steps. (**a**) Spinning photoresist on a silicon substrate. (**b**) Exposing the photoresist layer to define double spiral pattern. (**c**) Iron catalyst thin film deposition. (**d**) Lift-off patterning of the iron catalyst layer. (**e**) Aligned CN_x_CNT synthesis. (**f**) Assembly of microfluidic device, and (**g**) Operation principle of the microfluidic device.

We constructed this microdevice by integrating techniques of chemical vapor deposition (CVD) synthesis and microfabrication^38^. The process steps are shown in Fig. 2. We patterned iron thin films using the photoresist lift-off process. First, we spin-coated photoresists, LOR-5A, and SPR3012 in sequence at 4,000 RPM (Fig. 2a). Subsequently, we exposed the photoresist by an exposer (MA-6, SUSS contact aligner) to define a double spiral pattern (Fig. 2b). Next, we deposited iron catalyst thin films of 5 nm in thickness using an e-beam evaporator (Fig. 2c) and soaked it inside a solvent (Remover-PG, MicroChem) overnight (Fig. 2d). We then diced patterned substrates into a 1 cm × 1 cm device using a dicing saw. To grow CN_x_CNTs on individual devices, we used CVD with benzylamine as a precursor (Fig. 2e). The precursor was generated by an ultrasonic nebulizer and transported by argon/hydrogen gas into two furnaces in series at 825 °C with a flow rate of 2.5 L/min. On this patterned substrate, we grew CN_x_CNTs of 60 µm in height. Then, we built a microfluidic device by bonding a top cover made of PDMS (Fig. 2f). The mass of an assembled device was only 1.5 gram, which is three orders of magnitude lighter than a conventional centrifuge used for plasma extraction. This PDMS top cover was fabricated by micro-molding with fluidics accesses and a chamber of 50 µm in height. We defined the chamber dimensions by employing an SU-8 mold and by puncturing three fluidic access ports: one inlet and two outlets. The chamber height was slightly lower than the CN_x_CNT channel wall to achieve better sealing between PDMS and the top of the A-CN_x_CNTs. To enhance bonding, surfaces of both PDMS top cover and A-CN_x_CNT channel walls were treated with an oxygen plasma. Before processing the blood samples, we flushed microdevices by 0.5% Tween-20 and phosphate-buffered saline (PBS) sequentially.

## Results

### Characterization of a double spiral microdevice

We apply a homogeneous membrane (3D filter) of aligned CN_x_CNTs. This membrane has to be biocompatible, porous, and to exhibit controllable inter-tubular distances. We used scanning electron microscopy (SEM) and Raman spectroscopy to characterize the structural properties of our CN_x_CNTs.

**Figure 3:**
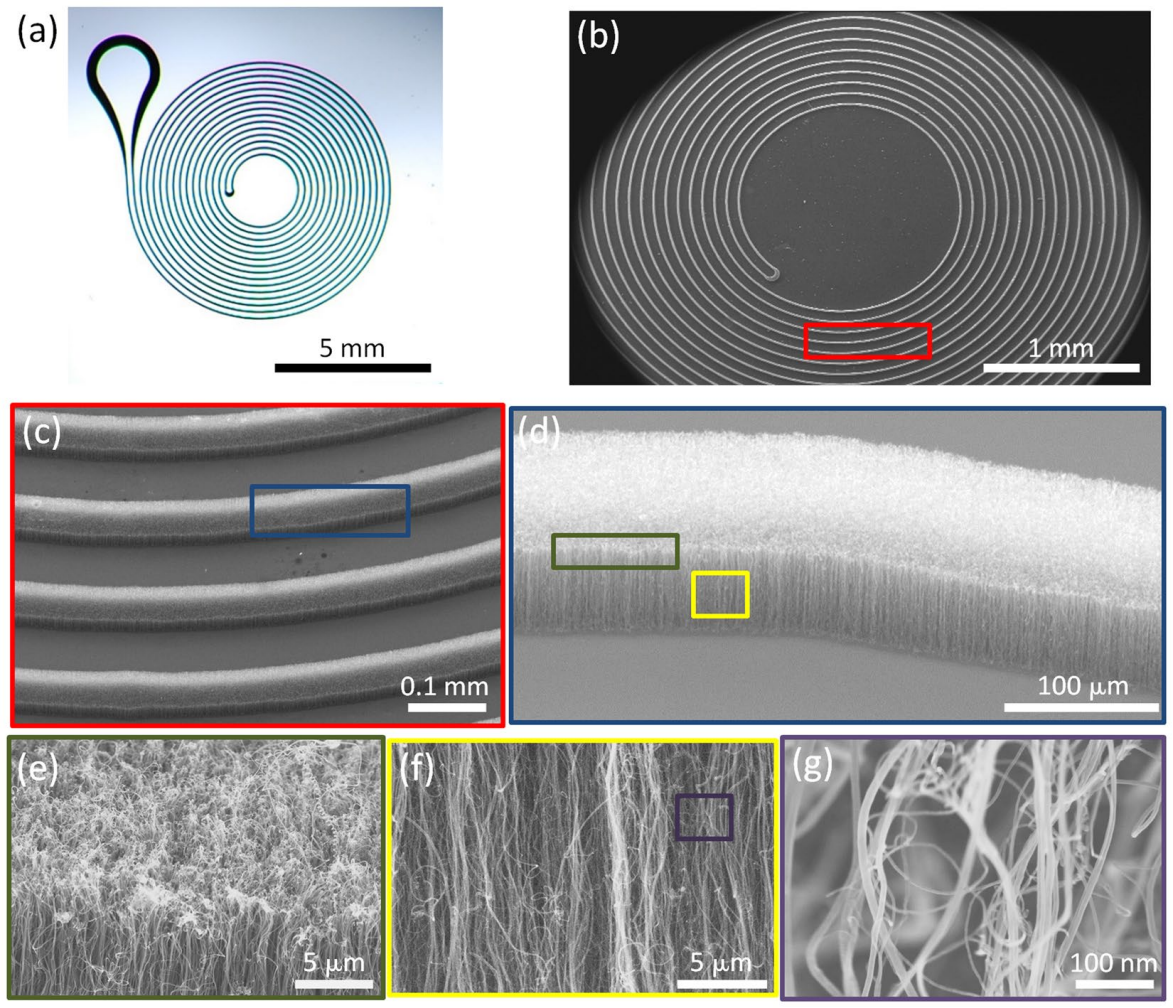
Optical and scanning electron microscopy (SEM) images of grown CN_x_CNT before assembling the microfluidic device with PDMS. (**a**) Top-view optical image of the CN_x_CNT grown on channel wall following a double spiral pattern. (**b**) SEM image of the CN_x_CNT porous channel walls. (**c**) Zoom-in SEM image of an individual CNT porous channel wall. (**d**) Top surface of a CN_x_CNT channel wall. (**f**) Cross-sectional view of CN_x_CNT arrays, and (**g**) Zoom-in SEM image showing individual CN_x_CNTs.

As explained above, the miniaturized microdevices consist of aligned CN_x_CNTs. Figure 3a shows one patterned microdevice after growing CN_x_CNTs for 30 minutes. Figure 3b confirms that CN_x_CNTs only grow on the pre-patterned iron thin film and form a microfluidic channel wall. This double spiral channel has a wall thickness of 100 µm and a channel width of 100 µm (Fig. 3c). Figure 3d depicts aligned CN_x_CNTs of uniform height (~60 µm). Figure 3e demonstrates that the height variation is below 5 µm. This particular array exhibits a ~80 nm inter-tubular distance (Fig. 3f,g). Controlling the inter-tubular distance is critical for allowing plasma and other smaller moieties such as proteins to pass through while blocking larger particles (>1 µm), including platelets and cells that are contained in blood.

**Figure 4:**
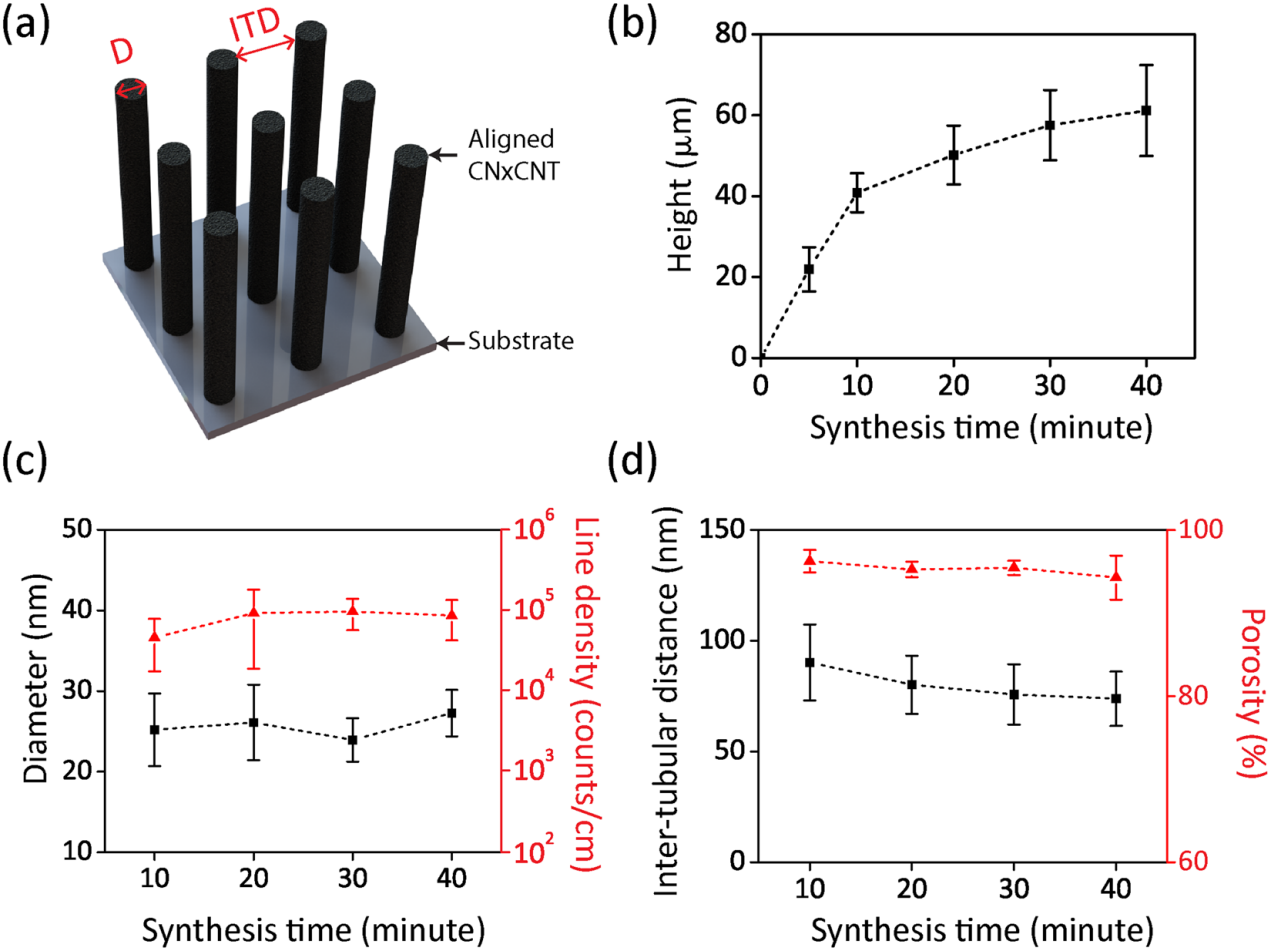
Characterization of A- CN_x_CNTs for different synthesis times. (**a**) Illustration of aligned CN_x_CNTs with labels showing the physical dimensions of diameter (D) and inter-tubular distance (ITD). (**b**) Height. (**c**) Diameter and Line density, and (**d**) Inter-tubular distance and porosity.

Controlling the height and inter-tube distances of CN_x_CNTs is very important for the design of our micro-fluidic device. In this context, we grew CN_x_CNTs for 10, 20, 30, and 40 minutes and measured the diameter, density, and inter-tubular distance (ITD) from cross-sectional SEM images (Fig. 4a). In Fig. 4b, the height of the aligned CN_x_CNTs increases with synthesis time. The height of CN_x_CNTs reaches 61.1 ± 10.1 µm after 40 minutes of synthesis. For nanotube diameter measurements, the images were taken under 6 × 10^4^ magnification. A total number of 200 CNTs were measured for each synthesis. For the density measurements, we counted the number of CNTs per unit length. Both the diameter and density of aligned CN_x_CNTs were independent of each synthesis experiment. The aligned CN_x_CNTs possess an average diameter of 26.5 ± 1.2 nm and a density of 1.7 × 10^9^ ± 7.4 × 10^8^ counts/cm (Fig. 4c). Similarly, we measured ITD of CN_x_CNTs to be 80.0 ± 7.3 nm (Fig. 4d). The ITD was measured from bottom sections of the CN_x_CNT arrays, where tubes were better aligned in the vertical direction compared to the upper sections^3,39^. Next, we employed a geometrical model to describe the dimensions of the CNT arrays^35^. This model assumes that the orientation of the aligned CN_x_CNT structure follows a cylindrical model with uniform density and diameter. For the model of cylindrical pillars, the bulk porosity of the cylindrical array is described below where ∅ is the porosity, P is the inter-tubular distance, and D is the diameter of the cylindrical pillar^40^:

1 $$\varnothing =1-\frac{{\rm{\pi }}}{4}\times \frac{{{\rm{D}}}^{2}}{{({\rm{P}}+{\rm{D}})}^{2}}$$

As shown in Fig. 4d, the porosity was calculated using density and diameter measurements extracted from Fig. 4c. The result showed that the porosity of aligned CN_x_CNTs reached average values as high as 93.8 ± 0.3%. In comparison with other types of porous membranes, this high porosity of the CN_x_CNTs can significantly increase the extraction efficiency (see below).

**Figure 5:**
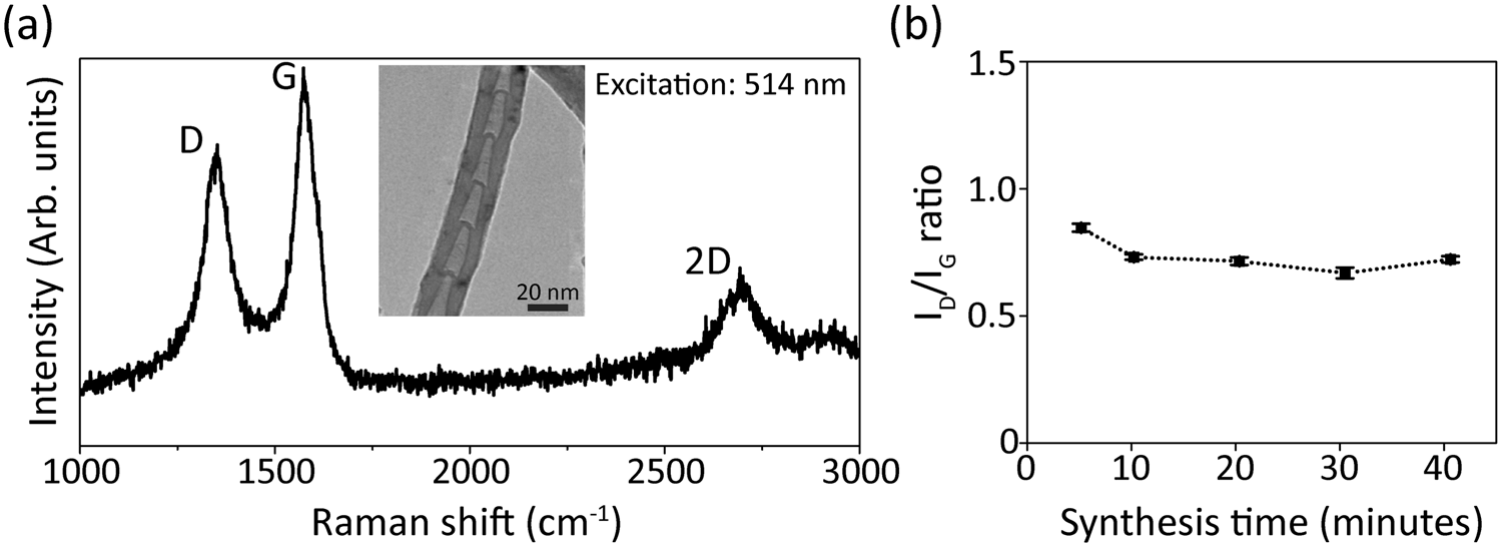
Raman characterization of CN_x_CNTs. (**a**) Raman spectra of aligned CN_x_CNTs. Inset is a transmission electron microscopy (TEM) image of an individual CN_x_CNT, and (**b**) Intensity ratio of the D- to G-band under different synthesis times.

Raman spectroscopy is a well-established tool to characterize CNTs in a non-destructive manner^41^. Raman spectroscopy measures the degree of crystallinity of CNTs, chirality in the case of single-walled nanotubes, defects, etc.^42,43^. In our study, we used Raman microscopy (using a Renishaw, InVia Raman microscopy) to characterize our synthesized CN_x_CNTs. Raman spectra were recorded using a 514 nm laser excitation for 30 seconds under 50X magnification. The laser power used for the measurements was 10 µW. The Raman spectrum showed the D-band centered at 1352 cm^−1^, G-band at 1578 cm^−1^, and D′-band at 2659 cm^−1^, respectively (Fig. 5a). The intensity ratio of D-band to G-band is *ca*. 0.7, a value significantly higher than that obtained for un-doped CNTs. Since the D-band is a second order feature originated by structural defects in CNTs, the high I_D_/I_G_ ratio suggests structural disorder, such as the presence of nitrogen dopants and vacancies within the tubes lattice (Fig. 5b)^44^. Furthermore, the characteristic bamboo-like morphology of CNTs shown in the inset of Fig. 5a provides a clear signature for nitrogen doping^29,45–47^. As indicated above, the successful incorporation of nitrogen dopants in the lattice is vital for enhancing the bio-compatibility of CNTs, but the exact mechanism of such enhancement requires further investigation^29,31,48^.

### Plasma extraction and albumin measurement

**Figure 6:**
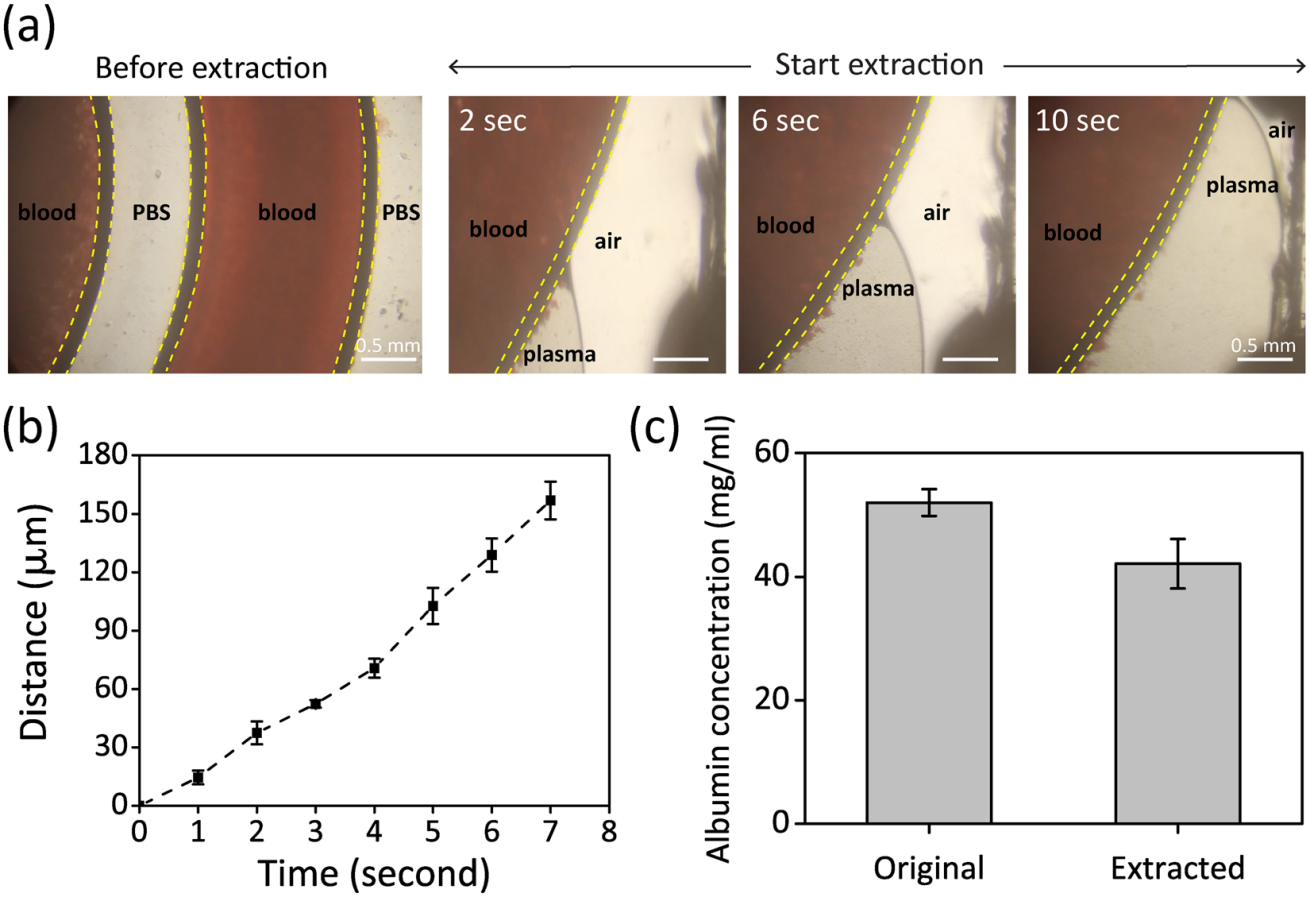
Top views of the blood transported inside the microdevice and characterization of the extraction rate and efficiency of albumin. (**a**) Bright field images of whole blood introduced inside a microdevice (before extraction) and time-lapsed images of plasma extraction (at the beginning of extraction). The yellow dot lines indicate the boundary of A-CN_x_CNT walls. (**b**) Measurements of extraction efficiency over time. (n = 4), and (**c**) Albumin concentration measured from original blood and the extracted plasma samples using our devices. (n = 6).

To characterize the plasma extraction performance, we measured the albumin concentration from different extractions (BCP Albumin Assay Kit #MAK 125, Sigma-Aldrich). Albumin is the most abundant protein inside plasma and serves as a biomarker of different diseases^49–52^. During extractions, we monitored blood flow in real-time under a bright-field optical microscope to track red blood cells. Blood samples were drawn from a healthy donor into 10-mL EDTA coated tubes (Vacutainer; Becton Dickinson). Samples in EDTA tubes were processed within 24 hours. Before processing blood samples, we wet a microdevice by flushing a surfactant (0.5% Tween-20, #P9416 Sigma-Aldrich) until all the air inside a microdevice was replaced. Subsequently, we introduced PBS with a rate of 100 µL/min for five minutes to wash off the residual surfactant inside the microdevices. After flushing, we turned on a vacuum source connected to the outlet and started removing the residual PBS inside the microdevice. We transported blood samples into a microdevice at a rate of 100 µL/min. As demonstrated in Fig. 6a, we observed that red blood cells were transported and confined inside a double spiral channel. Simultaneously, plasma diffused through A-CN_x_CNT channels; yet blood cells were still confined inside the double spiral channel without leaking or clogging. Time-lapse images shown in Fig. 6a reveal the initial stage of blood plasma is displacing the air inside the spiral channel at a speed of ~24 µm/sec (Fig. 6b). The filtrate plasma was then collected at outlet-ii. We measured albumin concentration using BCG Albumin Assay Kit (Sigma-Aldrich MAK124). The albumin concentrations of the original sample and outlet were 52.0 ± 2.1 mg/mL and 42.1 ± 4.1 mg/mL, respectively. Therefore, this device recovers 80.1 ± 5.4% of the albumin within the extracted plasma. The successful extraction of plasma is attributed to unique high porosity (>90%).

## Conclusions

We successfully constructed a microfluidic device with a porous channel wall consisting of aligned CN_x_CNTs. This porous channel exhibits a high porosity (>90%) with a nanometer scale inter-tubular distance (~80 nm). This channel separates micron-scale blood cells, such as leukocytes (diameter: 12~17 µm) and erythrocytes (diameter: 3~5 µm) from nano-scale biomarkers, such as albumin (diameter: ~5 nm). These aligned tubes allowed an effective separation of micron-sized particles within whole blood, with a recovery rate of ~80%, averaged from at least 20 devices. Also, this miniaturized device is disposable and three orders of magnitude lighter in weight than conventional centrifuges. This novel portable microdevice now allows point-of-care diagnostics by extracting plasma containing proteins of key biomarkers.

## Materials and Methods

### Fabrication of iron catalyst thin film and CNT growth

Detailed information is described in our previous report^34^. In short, the iron catalyst thin film was deposited by e-beam evaporation and further patterned by a lift-off process. The CN_x_CNT was synthesized by AACVD using Benzylamine as a precursor. The deposition was performed at 825 °C for 30 minutes, under argon and 15% hydrogen flow of 2.5 L/min.

### Raman characterization of CN_x_CNT

A Raman microscopy (Renishaw, InVia) with a 514 nm laser was employed. Spectra were acquired under a 50× objective lens for 30 seconds.

### Device assembly and experimental setup

As described in our previous study^34^, the PDMS mold was manufactured by using a commercialized kit (Sylgard 184, Dow Corning). Before bonding, RF oxygen plasma (M4L, PVA TePla Inc) was applied to activate both the PDMS and CN_x_CNT surfaces. After bonding, the microdevices were baked at 85 °C for four hours.
